# Electron cotunneling through doubly occupied quantum dots: effect of spin configuration

**DOI:** 10.1186/1556-276X-6-251

**Published:** 2011-03-23

**Authors:** Jian Lan, Weidong Sheng

**Affiliations:** 1 Department of Physics, Furan University, Shanghai 200433, PR China

## Abstract

A microscopic theory is presented for electron cotunneling through doubly occupied quantum dots in the Coulomb blockade regime. Beyond the semiclassic framework of phenomenological models, a fully quantum mechanical solution for cotunneling of electrons through a one-dimensional quantum dot is obtained using a quantum transmitting boundary method without any fitting parameters. It is revealed that the cotunneling conductance exhibits strong dependence on the spin configuration of the electrons confined inside the dot. Especially for the triplet configuration, the conductance shows an obvious deviation from the well-known quadratic dependence on the applied bias voltage. Furthermore, it is found that the cotunneling conductance reveals more sensitive dependence on the barrier width than the height.

## Introduction

Semiconductor quantum dots have been known for their excellent electronic properties, and hence become attractive candidates to realize quantum bits and related spintronic functions [1]. Such spintronic devices are based on a spin control of electronics, or more specifically, an electrical control of spin in spin-dependent transport through a semiconductor quantum dot [2]. A good understanding of properties of an electron spin in quantum dots, in particular, its control and engineering in the electron scattering and transport, is therefore the key to the success of the perspective applications in spintronics.

In the Coulomb blockade regime where the sequential tunneling transport is greatly sup-pressed, electron conduction is dominated by cotunneling processes [3-5]. The cotunneling transport can be either elastic if the transmitting electron leaves the dot in its ground state, or inelastic if the applied bias exceeds the lowest excitation energy and the dot is left in an excited state. Quantum dots are usually modeled as simple semiclassical capacitors to explain Coulomb blockade effect and spin-related transport phenomenon [6]. Although conventional approach like Green's function or master equation combined with Hubbard model has been quite successful in both the sequential tunneling and cotunneling regimes [7], there have been several theoretical attempts on dealing directly with the many-body Hamiltonian to study the few-electron transport problem recently [8,9]. However, it still presents a great challenge to obtain a fully quantum mechanical solution for cotunneling of electrons through a quantum dot that is beyond the semiclassic framework of phenomenological models.

## Model and Method

**Figure 1:**
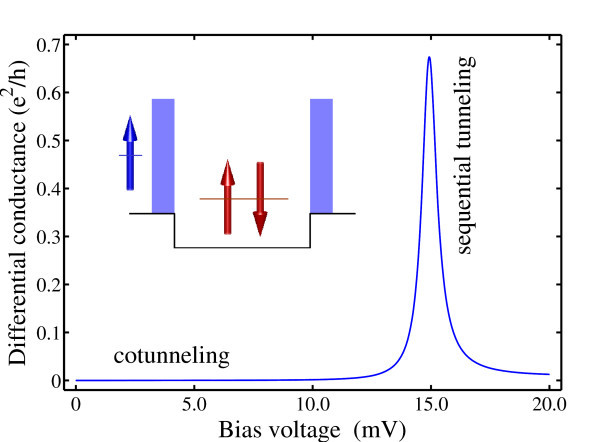
**Differential conductance for an electron transporting through a doubly occupied quantum dot calculated as a function of the applied bias voltage**. Inset: a schematic view of the model system.

An approach beyond the conventional phenomenological models is presented to directly solve the many-body Hamiltonian in the electron transport through a few-electron system without applying any approximations to the electron-electron interaction. A schematic view of our model system is shown in the inset of Figure 1. The quantum dot is modeled as a one-dimensional double-barrier structure, each barrier has a height of 50.0 meV and width of 5.0 nm, and the potential well in-between has a width of 30.0 nm and depth of -15.0 meV below the bottom of the outside barriers. Considering the penetration of the confined states into the barriers, we have placed two buffer layers on the left and right sides of the system.

The quantum dot is assumed to be doubly occupied. Electron transmitting through such a system involves three electrons, the incident one and two confined ones. The Hamiltonian of these interacting electrons is given by(1)(2)

where *V*_QD_(*x*) is the potential defining the device structure, and the effective mass of electrons (m*) and dielectric constant (*ε_r_*) are chosen to be the values for GaAs. In order to obtain a fully quantum mechanical solution for electron transport through a doubly occupied system, we first compute the energy levels of the two interacting electrons which are governed by the following Hamiltonian,(3)

The one-dimensional problem of two interacting electrons can be mapped into that of a single electron in an effective two-dimensional potential as follows:(4)(5)

By imposing appropriate symmetry conditions, the *exact *energy levels as well as the wave functions of two interacting electrons can be calculated by a finite-difference method. By calculating the Coulomb matrix elements [10], one can estimate the proportion of the correlation energy in the energy of a two-electron state. For the example of the ground state, we have  which is larger than 1.42meV, the exchange energy between the ground and first excited single-particle state *U*_1221_.

For the few-particle scattering problem as shown in Figure 1 the wave function of the *three *interacting electrons in the incident terminal is given by(6)

with *φ_m_*(*x*) the *m*th two-particle state being confined in the quantum dot, and *k *being the wave vector of the incident electron. *k_m _*satisfies(7)

with *E *being the energy of the incident electron. Similarly, on the outgoing side, we have(8)

The interchange symmetry for states with identical particles requires the following transformation for both *ψ*_in_(*x*_1_, *x*_2_, *x*_3_) and *ψ*_out _(*x*_1_, *x*_2_, *x*_3_) for the spin configuration as shown in Figure 1(9)(10)

The three-particle scattering problem can now be mapped into the scattering of a single electron in the following effective three-dimensional potential:(11)

The wave function in the scattering area together with the unknown coefficients *t_m _*and *r_m _*is solved by using a quantum transmitting boundary approach [11] which is recently generalized to the few-particle regime [12]. The finite-difference algorithm results in a system of linear equations for *N*^3 ^unknown variables where *N *is the number of the mesh points along each dimension. Here, we find that converged result can be achieved for *N *= 50 using the conventional bi-conjugate gradients iteration method with the incomplete LU factorization as a preconditioner. It is noted that all the electron-electron interactions including the correlation and exchange effects are fully incorporated in the calculation. The probability of an electron transmitting through the device while leaving the others in the *m*th confined state in the quantum dot is described by the partial transmission *T_m_*(*E*) which is given by(12)

where *T*_1 _being for the usual elastic scattering process, *T_m _*(*m *> 1) describes the probability of the inelastic scattering process in which the energy of the outgoing electron is smaller than that of the incident one. The total transmission is hence given by(13)

The differential conductance is therefore given by *G*(*V*) = *T *(*V*)*e*^2^/*h *where *eV *= *E *with *V *being the bias voltage. Since we are dealing with electron of definite spin, the spin degeneracy does not appear in the Landauer formula.

## Result and Discussion

The differential conductance calculated as a function of the applied bias voltage has been shown in Figure 1. Around 15.0 mV are seen sequential tunneling peaks where the energy of the incident electron combined with that of two confined ones happens to be aligned with the ground state level of three interacting particles localized inside the dot.

**Figure 2:**
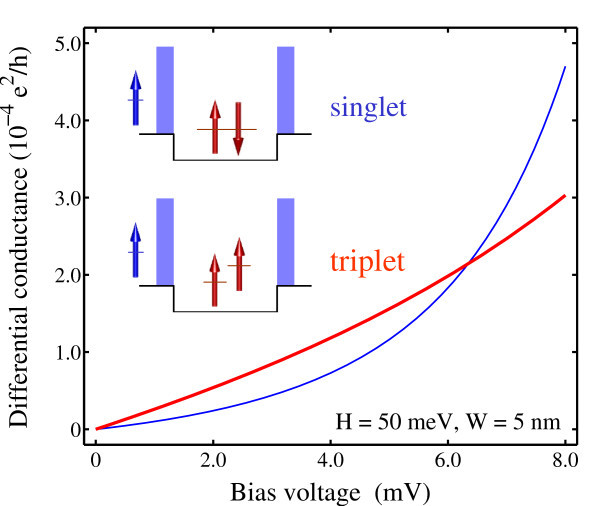
**Cotunneling conductance calculated as a function of the applied bias voltage for the dot occupied by a singlet (thin lines) and triplet (thick lines)**.

In the Coulomb blockade regime where the sequential tunneling transport is greatly suppressed, e.g., when *V *< 10 mV in Figure 1 electron conduction is dominated by cotunneling processes [4,5]. Figure 2 plots the conductance of electron cotunneling through a dot occupied by a singlet and triplet. Since cotunneling current is generally several orders of magnitude smaller than the sequential tunneling, we have to set the precision for the iterative solver to be 10^-6 ^to obtain reliable result. For the case of a singlet in the dot, it is seen that the cotunneling conductance closely follows the well-known quadratic dependence [13] on the applied bias voltage. The cotunneling conductance in the case of a triplet is found not only generally larger than the singlet but also deviates obviously from the quadratic dependence. Actually, the conductance is seen to be almost linear with the bias voltage with a very small quadratic term. For comparison, the conductance of electron cotunneling through a singly occupied quantum dot exhibits very little dependence on the spin configuration of the incident and confined electrons [14]. Furthermore, it exhibits much less deviation from the quadratic dependence than the cotunneling conductance for the triplet configuration.

It shall be noted that the model used in this study is a one-dimensional system. For such a simplified model, both the density of states of the incoming electrons and electron-electron interactions inside the quantum dot are dierent from those in the conventional two-dimensional lateral structures, which could at least partially account for the deviation of the cotunneling conductance from the quadratic dependence.

**Figure 3:**
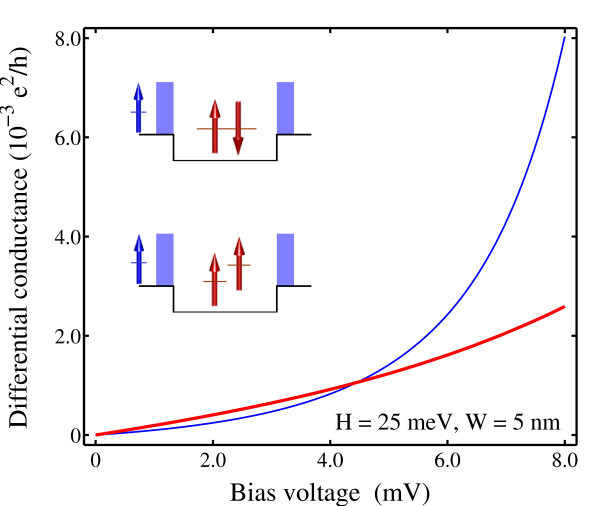
**Cotunneling conductance calculated as a function of the applied bias voltage for the dot occupied by a singlet (thin lines) and triplet (thick lines)**. The height of barriers is reduced to 25 from 50 meV.

As conventional phenomenological models do not usually give the dependence of the cotunneling conductance on the structural parameters, it is interesting to see how the conductance changes with the barrier width and height. Figure 3 shows the result obtained for a dot of reduced height of barriers. It is seen that the cotunneling conductance increases by more than one order of magnitude as the height of barriers is reduced by half. With lower barriers, the sequential tunneling peak would have red shift and may account for larger influence on the cotunneling conductance at the low energy end. However, the sequential tunneling peak for the triplet occupation is beyond 25 meV with lower barriers and hence shall have very little effect on the cotunneling conductance for the energy below 8 meV. Nevertheless, the cotunneling conductance in the case of triplet is found to increase with even greater amplitude than singlet. Therefore, it is the reduced height of barriers instead of the indirect influence of the sequential tunneling peak that accounts for the largely enhanced cotunneling conductance.

**Figure 4:**
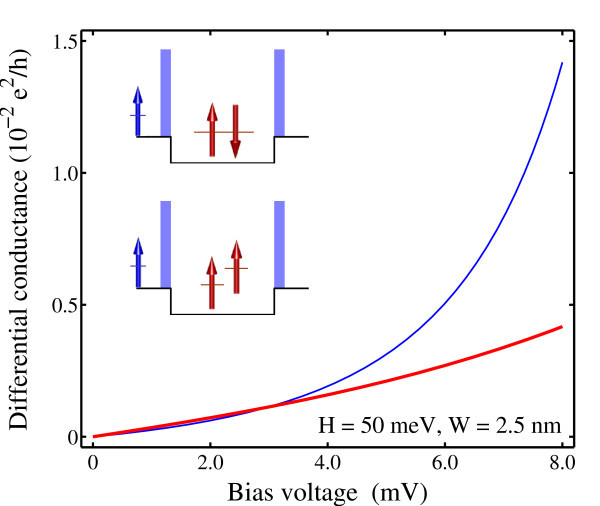
**Cotunneling conductance calculated as a function of the applied bias voltage for the dot occupied by a singlet (thin lines) and triplet (thick lines)**. The width of barriers is reduced to 2.5 from 5.0 nm.

Let us see next how the cotunneling conductance depends on the barrier width. Figure 4 plots the cotunneling conductance calculated as a function of the applied bias voltage for the dot of thinner barriers. With the width of barriers reduced by half, the cotunneling conductance is seen to be almost twice as larger as that with lower barriers. It can therefore be concluded that the dependence of the cotunneling conductance is more sensitive on the barrier width than the height.

With lower or thinner barriers, it is seen that the cotunneling conductance exhibits greater difference between the cases of singlet and triplet occupations. The cotunneling conductance in the presence of singlet is found to increase more rapidly with energy than in the presence of triplet.

## Conclusion

To summarize, we have presented a microscopic theory of electron cotunneling through doubly occupied quantum dots in the Coulomb blockade regime beyond the semiclassic framework of phenomenological models. The cotunneling conductance is obtained from a fully quantum mechanical solution to the transport problem of three interacting electrons in a one-dimensional quantum dot by using a quantum transmitting boundary method without any fitting parameters. We have revealed that the conductance exhibits strong dependence on the spin configuration of the electrons confined inside the dot. Especially for the triplet configuration, we find that the cotunneling conductance shows an obvious deviation from the well-known quadratic dependence on the applied bias voltage. Furthermore, the cotunneling conductance has been shown to have more sensitive dependence on the width of the barriers than on the height.

## Competing interests

The authors declare that they have no competing interests.

## Authors' contributions

JL carried out numerical calculations as well as the establishment of theoretical formalism and drafted the manuscript. WDS conceived of the study, and participated in its design and coordination.
